# A Case of Paediatric Anti-Glomerular Basement Membrane Disease Associated with Thrombotic Thrombocytopenic Purpura

**DOI:** 10.1155/2022/2676696

**Published:** 2022-08-27

**Authors:** Joseph McAllister, Pradeep Nagisetty, Kay Tyerman

**Affiliations:** Paediatric Nephrology, Leeds Childrens Hospital, Leeds LS2 9NS, West Yorkshire, UK

## Abstract

Anti-GBM disease is a rare vasculitis that causes rapid progressive glomerulonephritis and pulmonary haemorrhage. It is usually an adult diagnosis with isolated paediatric cases reported. Thrombotic thrombocytopenic purpura (TTP) is a rare thrombotic microangiopathy mainly affecting adults that causes multiorgan ischaemia, microangiopathic haemolytic anaemia, and thrombocytopenia. We present the first paediatric case of concurrent anti-GBM disease and TTP. A 14-year-old boy presented with acute kidney failure and severe pulmonary haemorrhage due to anti-GBM disease, confirmed on auto-antibody testing. There was thrombocytopenia and moderately low ADAMTS13 activity suggestive of TTP. The renal prognosis was poor with a need for dialysis. He was severely unwell with pulmonary haemorrhages requiring the use of extracorporeal membrane oxygenation (ECMO). His disease was treated with corticosteroids, plasma exchange (PEX), rituximab, and cyclophosphamide, resulting in remission. Anti-GBM disease is rare in children but should be considered in those presenting with acute kidney injury, particularly where there has been exposure to pulmonary irritants. An aggressive presentation warrants aggressive treatment with methylprednisolone, PEX, and cyclophosphamide. Rituximab may benefit patients that have concurrent TTP. TTP may exacerbate pulmonary disease, but complete respiratory recovery is possible. Disease relapse is rare in the paediatric age group, and these patients are candidates for kidney transplantation.

## 1. Introduction

Our case report describes a paediatric patient with anti-GBM disease and concurrent anaemia and thrombocytopenia attributed to thrombocytopenic thrombotic purpura (TTP). Anti-GBM disease is a serious and very rare small vessel vasculitis that occurs in 1–2 per million people per year [1]. It is seen more frequent in white Caucasians, females, and people in their third and sixth decades [2]. It is an extremely rare disease in paediatric patients, with data limited to case reports. In our tertiary paediatric nephrology center no cases of anti-GBM disease had been recorded over the previous two decades.

The aetiology of the anti-GBM disease has not been fully determined [3]. Cigarette smoking correlates positively with the incidence of pulmonary haemorrhage in anti-GBM disease and has been hypothesised as a potential trigger [4, 5]. Hydrocarbon inhalation appears to be another important environmental factor [5, 6]. Infectious triggers may also play a role, and Goodpasture's has been reported following cases of influenza A [7] and Burkholderia infection [8]. In patients presenting with Goodpasture's disease and pyrexia, infection was found in 78% of patients [9]. Genetic susceptibility is important and anti-GBM disease is frequently associated with HLA-DRB1 alleles [10]. The key clinical features of the anti-GBM disease are rapidly progressive glomerulonephritis and pulmonary haemorrhage. However, association with thrombotic thrombocytopenic purpura (TTP) and microangiopathic haemolytic anaemia has been described in adult patients [11, 12].

TTP is a rare thrombotic microangiopathy that annually affects 10 per million people. In paediatrics, the diagnosis is even rarer, and childhood TTP accounts for just 10% of cases [13]. The underlying pathology is a severe deficiency in ADAMTS13 activity caused by autoantibodies to ADAMTS13 or, rarely, recessively inherited genetic mutations of ADAMTS13 (Upshaw–Schulman syndrome). ADAMTS13 cleaves the A2 subunit of the von Willebrand factor (VWF) [14], and deficiency of ADAMTS13 results in abnormally large prothrombotic VWF polymers [23]. This results in TTP and the findings of multiorgan ischaemia, microangiopathic haemolytic anaemia, and severe thrombocytopenia [15]. It is a serious and life-threatening disease in its own right, with a mortality rate of 10–20% [13].

## 2. Case Presentation

A 14-year-old boy presented to his local emergency department with a three-week history of illness. His symptoms were diarrhoea, vomiting, lethargy, epistaxis, haematuria, and oliguria for two days. At presentation, he had a cardiovascular shock and an intravenous bolus of 20 ml/kg of 0.9% sodium chloride was administered. He was treated for sepsis with intravenous cefotaxime and metronidazole. Urgent blood testing revealed metabolic acidosis and severe acute kidney injury (AKI) with hyperkalaemia (urea 151 mmol/L, creatinine 4120 umol/L, K 8.7 mmol/L). Electrocardiogram changes of hyperkalaemia were evident. The full blood count showed anaemia (Hb 85 g/L, platelets 181 10*∗*9/L, WCC 19.7 10*∗*9/L).

Emergency management of hyperkalaemia was administered and he commenced haemodiafiltration via a femoral simple central venous dialysis catheter. He was transferred to a tertiary paediatric intensive care unit (PICU) for continuous venovenous haemodialysis (CVVHD). Renal ultrasound was unremarkable and further workup of his kidney injury was undertaken.

**Figure 1:**
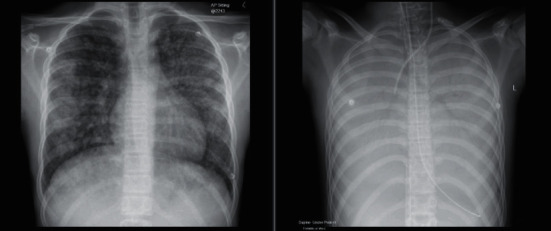
Early and late chest radiograph's demonstrating pulmonary haemorrhage.

He was transferred to the regional paediatric nephrology unit after two days. At this point, he appeared alert and well, was normotensive at 118/70 mmHg, and self-ventilating in air. Repeat bloods on the ward showed anaemia and thrombocytopenia (Hb 65 g/L, Plt 43 10*∗*9/L). He continued on intermittent haemodialysis, pending the results of a nephritis screen and an investigation of possible haemolytic uraemic syndrome. Over the next 24 hours, he experienced respiratory deterioration requiring intubation and ventilation. This was initially attributed to pulmonary oedema, but during intubation there was evidence of massive pulmonary haemorrhage (shown in Figure 1). He was resuscitated with blood products (6 units of red cells, 3 units of fresh frozen plasma, 2 units of cryoprecipitate, and 2 units of platelets). His coagulation screen was normal (PT 12s, APTT 25.7s, INR 1.0, fibrinogen 3.4 g/L). The diagnosis of anti-GBM disease was suspected with clinical evidence of nephritis and pulmonary haemorrhage and he commenced intravenous methylprednisolone and plasma exchange (PEX).

Oxygenation was challenging despite high-frequency oscillatory ventilation (HFOV). His stay was also complicated by hypertension. Extracorporeal membrane oxygenation (ECMO) was commenced and continued for 55 hours at a quaternary ECMO center. His anti-GBM titer returned at >8 antibody index (AI) (reference range <0.99 AI) confirming the diagnosis of anti-GBM disease.

After repatriation to the PICU, he was noted to have persistent anaemia and thrombocytopenia despite transfusions of red cells and platelets. There had been no further pulmonary haemorrhage and examination did not reveal any other bleeding. There was no splenomegaly. Anaemia was attributed to haemolysis, and this was supported by raised serum bilirubin and LDH peaking at 155umol/L (2–21) and 1929 iu/L (120–246), respectively, and low serum haptoglobin of 0.07 g/L (0.50-2-00). Red cell fragments were detected on the blood film. Heparin-induced thrombocytopenia (HIT) secondary to heparin administration during plasma exchange was considered and excluded following a negative IgG platelet factor 4/heparin test. Atypical haemolytic ureamic syndrome was considered, and genetic testing was later found to be negative. ADAMTS13 activity was low at 26.7 IU/dL (reference range 52.0–149.0 IU/dL) and the ADAMTS13 inhibitor was negative. This raised the suspicion of concurrent TTP.

In planning further therapy, the kidneys were not expected to recover and we hoped to avoid cyclophosphamide and its side effects. Rituximab was initiated to treat both anti-GBM disease and TTP. During his first rituximab infusion, the patient developed high pyrexia, tachycardia, and testicular pain, and the treatment was abandoned. On the day of rituximab treatment, he received a platelet transfusion for a platelet count was 9 10*∗*9/L. Over the following three weeks, there was a gradual increment in platelet count to greater than 150 10*∗*9/L.

Our patient had a third PICU admission after his 16^th^ session of PEX, secondary to a pulmonary haemorrhage and a generalised tonic-clonic seizure attributed to posterior reversible encephalopathy syndrome. He required intubation, paralysis, sedation, and high-frequency oscillatory ventilation with nitric oxide. Initially, he was extremely unstable, with episodes of bradycardia requiring chest compressions, and hypoxaemia prompting serious consideration of his risk of death. Intravenous cyclophosphamide was commenced whilst in PICU and the anti-GBM levels improved (Regimen: 500 mg/m2 on day 0; 750 mg/m2 on days 16, 47, and 77) providing hope that his respiratory status would improve. After 28 days of ventilation, he was successfully extubated and moved to high dependency. He appeared neurologically intact but with significant loss of lean tissue mass, weighing 32.3 kg, down from 44.4 kg at admission.

**Figure 2:**
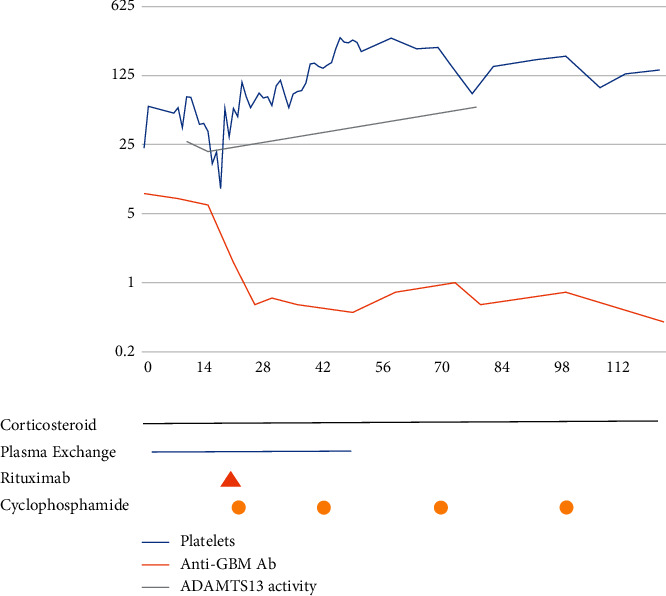
Logarithmic graph demonstrating platelet levels, anti-GBM antibody levels, ADAMTS13 activity alongside immunosuppressive therapy.

PEX was continued for a total of 33 sessions. Prednisolone was gradually reduced (by 5 mg every two weeks to 10 mg daily, then 10 mg alternate days, 5 mg alternate days, and then stopped) and then stopped over a four-month period. The anti-GBM antibody and the ADAMTS13 levels normalised within 3 months of diagnosis, at 0.80AI and 59.9 IU/dL, respectively (shown in Figure 2). The patient has defied all expectations and made a full respiratory recovery. One year after his presentation, he continues on in-centre haemodialysis three times a week and is progressing toward renal transplantation. Anti-GBM levels remain within the normal range.

## 3. Discussion

This was a very challenging case of anti-GBM disease and TTP. The association between anti-GBM disease and microangiopathic haemolytic anaemia or TTP is increasingly recognised after being reported by Stave et al. (1984) [11]. Our literature review identified a further eight case reports of thrombotic microangiopathy in adult patients with anti-GBM disease (shown in Table 1). Our case appears to be the first reported incidence of TTP and anti-GBM disease in a paediatric patient. Our case also stands out because of the extreme severity of nephritis and pulmonary disease.

40–60% of patients with anti-GBM disease present with both rapidly progressive glomerulonephritis and pulmonary haemorrhage [3]. Patients who smoke, as in this case, are at an increased risk of pulmonary haemorrhage [4]. The combination of TTP with anti-GBM-associated pulmonary haemorrhage may have potentiated our patients' pulmonary haemorrhage via deranged coagulation, already disrupted by plasma exchange therapy. Despite this, complete pulmonary recovery was achieved once remission was achieved.

Patients who require dialysis have a minimal prospect of renal recovery with 8% renal survival at 1 year [3]. Avoiding cytotoxic medication may be appropriate in these circumstances, and consideration of reproductive health and malignancy risk in teenagers is important. However, our experience suggests that in patients with severe and life-threatening pulmonary haemorrhage cyclophosphamide may induce disease remission more readily than rituximab.

The diagnosis of TTP was made with input from the tertiary paediatric haematology service. Our patient's ADAMTS13 was 26.7 IU/dL and he was ADAMTS13 inhibitor-negative. Although this was a modestly low ADAMTS13 level for TTP, the test was performed ten days after the commencement of steroids and PEX. PEX is known to increase ADAMTS13 activity in patients with inhibitor negative idiopathic TTP [23]. It is possible that the absence of the ADAMTS13 inhibitor is also attributable to PEX. The normalisation of the ADAMTS13 level to 59.9 IU/dL following treatment is suggestive of acquired TTP, as patients with the recessive Upshaw-Schulman syndrome continue to have severely reduced ADAMTS13 activity when well [15].

Following recovery, the patient has not relapsed during follow-up for over one-year allowing renal transplantation to be considered. Relapse in the anti-GBM disease is very rare (<3%) but may be associated with ongoing exposure to pulmonary irritants and ANCA positivity [3]. At least 40% of patients with autoimmune TTP experience at least one relapse [23]. Without the ADAMTS13 inhibitors, patients rarely relapse [13].

In conclusion, although rare in the paediatric age group, anti-GBM disease should be considered in children and young people presenting with acute kidney injury, particularly where there is a history of known risk factors. An aggressive presentation warrants aggressive treatment with methylprednisolone, PEX, and cyclophosphamide. Rituximab may have a beneficial therapeutic effect in patients with features suggesting associated TTP. Despite requiring ECMO support and prolonged periods of ventilation, a full pulmonary recovery is possible and disease remission achievable, allowing future kidney transplantation.

## Figures and Tables

**Table 1:** Summary of reports of concurrent anti-GBM disease and thrombotic microangiopathy.

First author	Year	Cohort size	Age	Sex	Hb (g/L)	Schistocytes	Platelet count (10*∗*9/L)	Biopsy confirmed TMA	ADAMTS13	ADAMTS13 inhibitor	Treatment	Outcome
Stave [11]	1984	6						Yes				
Stallworthy [16]	2006	1	64	Male	65	Present	42	No			PEX	Spontaneous remission over 3 months
Terryn [12]	2007	1	37	Male	77	Present	87	No			PEX Corticosteroids	Partial remission
Gowrishankar [17]	2009	1	38	Male	68	Present		Yes			PEX 9 sessions Corticosteroids	
Torok [18]	2010	1	43	Male	60	Present	35	No	Low (17)	Negative	PEX Corticosteroids Cyclophosphamide	Remission after 10 weeks
Vega-Carbrera [19]	2013	1	27	Male	100	Present	60	Yes	Low (<1%)	Present	PEX Corticosteroids Cyclophosphamide Rituximab Immunoglobulin	Remission within 5 weeks
Yu [20]	2017	1	41	Female	55	Present	28	Yes	Normal		PEX Corticosteroids	Remission
Alirezaei [21]	2018	1	25	Male	62	Present	44	Yes	Normal		PEX Corticosteroids Cyclophosphamide Rituximab	Remission within 1 month
Micarelli [22]	2020	1	71	Female	95	Present	13	No	Low (64)	Negative	PEX Corticosteroids Cyclophosphamide	Improvement in platelet count after 5 days

*∗*PEX = Plasma exchange. Empty cells represent unreported data.

## Data Availability

All data generated and analysed for this report are included in this article. Further enquiries can be directed to the corresponding authors.
